# Changes in Testing and Treatment Methods in Osteoporosis Care

**DOI:** 10.1155/2024/9629891

**Published:** 2024-04-17

**Authors:** Takashi Nagai, Koji Ishikawa, Koki Tsuchiya, Soji Tani, Yusuke Dodo, Yusuke Oshita, Keizo Sakamoto, Nobuyuki Kawate, Yoshifumi Kudo

**Affiliations:** ^1^Department of Rehabilitation Medicine, Showa University School of Medicine, Tokyo, Japan; ^2^Department of Orthopaedic Surgery, Showa University School of Medicine, Tokyo, Japan; ^3^Department of Orthopaedic, Showa University Northern Yokohama Hospital, Kanagawa, Yokohama, Japan

## Abstract

Osteoporosis treatment plays a crucial role in preventing fractures, particularly in bedridden patients. We conducted a questionnaire survey presenting hypothetical clinical cases in 2015 and 2020 to investigate trends over a 5-year period. The target population included physicians working in clinics and hospitals within our neighbourhood. The cases were presented, and the questionnaire was administered in a confidential format. The orthopaedic surgeons were matched for age and practice, resulting in 74 cases being included in the analysis. Comparing the 2015 and 2020 results, we observed a notable increase in physicians who would perform “bone mineral density measurements of the lumbar spine and hip.” Furthermore, there was a significant rise in the percentage of respondents willing to test for bone metabolic markers, such as serum type I collagen cross-linked N-telopeptide (NTX), procollagen I N-terminal propeptide (P1NP), and tartrate-resistant acid phosphatase 5b (TRACP-5b). Regarding therapeutic agents, bisphosphonates decreased in usage, whereas parathyroid hormone and romosozumab witnessed an increase. In conclusion, the percentage of physicians requesting bone mineral density measurements of the lumbar spine and hip increased over the five-year period. In addition, more physicians chose to utilise bone metabolic markers due to their ease of measurement through blood tests and reduced diurnal variation. Finally, there was a marked trend towards the administration of drugs capable of rapidly and effectively increasing bone mineral density at an early stage of treatment.

## 1. Introduction

Osteoporosis can occur in both men and women but is more likely to occur in women and is estimated to affect 200 million women worldwide, or about one tenth of 60-year-old women, one fifth of 70-year-old women, two fifths of 80-year-old women, and one third of 90-year-old women [1]. It is estimated that 1–1.5 million people in Japan experience vertebral fractures annually and approximately 100,000–150,000 people experience femoral neck fractures [2]. Therefore, the treatment of osteoporosis has become important in daily practice for fracture prevention in bedridden patients [3].

The guidelines for the prevention and treatment of osteoporosis were revised in 2015 [4]. Each drug has a recommended category based on evidence, with eldecalcitol, bisphosphonates, selective oestrogen receptor modulators (SERMs), parathyroid hormone (PTH) products, and denosumab recommended for their effectiveness in increasing bone mineral density and reducing lumbar vertebral and femoral neck fractures [4].

Since 2015, zoledronate has become insurable for the treatment of osteoporosis, followed by romosozumab in 2019. In addition, hydroxyvitamin D (25(OH)D) has been used for osteoporosis testing since 2018. It is up to the attending physician to decide which drug to use and which tests to perform. In other words, options were expanded for the attending physician.

We conducted our first survey in 2015 to determine how the choice of testing methods and therapeutic agents in osteoporosis practice differs between hospital- and clinic-based orthopaedic surgeons [5]. The second survey was conducted in 2020 to determine how testing and therapeutic agents in osteoporosis care have changed over the past five years. This study aimed to understand the differences between the attitudes of hospital- and clinic-based orthopaedic surgeons to facilitate collaboration between multiple hospitals as well as between hospitals and clinics. This study compared the results of the 2015 questionnaire with those of the 2020 questionnaire, which were limited to orthopaedic surgeons, using a propensity score analysis. To the best of our knowledge, this is the first report of an attitude survey among physicians treating osteoporosis using hypothetical cases.

## 2. Materials and Methods

### 2.1. Subjects and Methods

An anonymous questionnaire was sent to orthopaedic surgeons working in clinics and hospitals in the Tokyo area.

**Figure 1:**
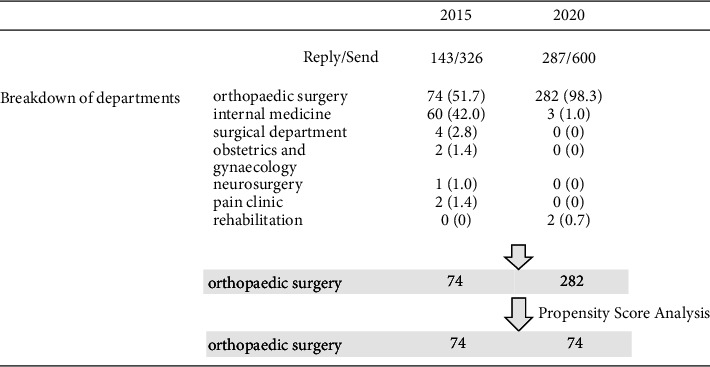
The protocol. A total of 143/326 responses were obtained in 2015, and 287/600 responses were obtained in 2020. From these, we matched orthopaedic surgeons by age and workplace setting and selected 74 cases in 2015 and 74 in 2020 for analysis.

Respondents were sent a case description, a preliminary questionnaire, a required questionnaire, and a response form and asked to return only the response form in the enclosed return envelope. The questionnaire presented a single case and allowed each person to choose from a list of tests and treatments they would perform, if any (Figure 1).

A propensity score analysis was performed, matching the respondents' age and the hospital- and clinic-based orthopaedic surgeons, and the cases were selected so that the ratio of the number of respondents in 2015 and 2020 would be 1 : 1.

### 2.2. The Presented Case

The hypothetical case presented in this study reflects a specific set of conditions based on our hypothetical scenario: early 70s, mild loss of bone mineral density, diabetes mellitus and possible deterioration of bone quality, no complaints of pain, and a history of a femoral neck fracture in the mother.

### 2.3. Case

The hypothetical case is that of a 72-year-old woman with the following characteristics: a height of 154 cm, a weight of 55 kg, a body mass index (BMI) of 23.2 kg/m^2^, and a thin build during her youth.

Menarche occurred at age 14 and spontaneous menopause at age 50.

No history of smoking or alcohol use.

Family history: her mother had a history of treatment for a proximal femur fracture.

Current history: ten years ago, a physical examination revealed hyperglycaemia, and a 75 g oral glucose tolerance test revealed diabetes mellitus. Since then, she has been treated with diet, exercise, and alpha-glucosidase inhibitors and has been improving and responding well to treatment.

There was no nephropathy, neuropathy, or retinopathy. She is on a diet therapy for dyslipidaemia.

Since her mother had a history of proximal femur fracture, bone mineral density measurements and blood tests were performed.

Blood biochemistry results: haemoglobin A1c: 7.2% (National Glycohemoglobin Standardisation Programme value) (normal value: 4.3–5.8), total cholesterol: 160 mg/dL, low-density lipoprotein cholesterol: 145 mg/dL, and high-density lipoprotein cholesterol: 60 mg/dL.

Bone mineral density: 1/3 radius (young adult mean 65%).

10-year risk of major osteoporotic fracture in the Fracture Risk Assessment Tool (FRAX): 20%

### 2.4. Questions

**Figure 2:**
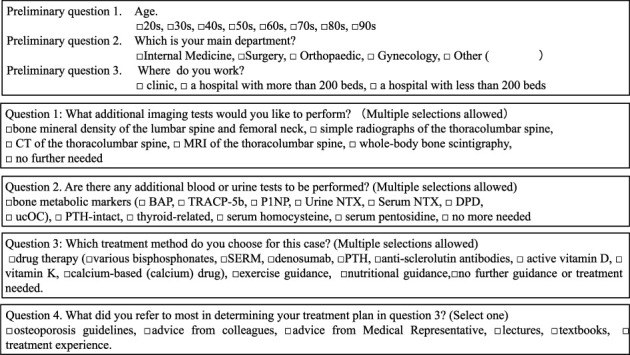
The questionnaire used in this study. The questionnaire asked three preliminary questions, additional imaging tests, blood and urine tests, treatment methods, and what was used as a reference in deciding on treatment methods.

Preliminary questions about age, main medical specialties, and work schedule were introduced in the questionnaire. Moreover, questionnaires asked about additional imaging tests, additional blood tests, urinalysis, treatment options, and what was most helpful in deciding on a treatment plan. The questions are shown in Figure 2.

### 2.5. Ethical Considerations

All research was properly conducted in accordance with the Declaration of Helsinki. Questionnaires were returned to the researcher if the surgeon agreed, after clearly stating in writing in advance that the responses may be published in a paper. Since responses were provided anonymously and the presented hypothetical cases were created by us and not based on real patients, ethical considerations regarding patient confidentiality and privacy were not applicable.

### 2.6. Statistical Analysis

Statistical analyses were performed using StatFlex (ver. 7.0.8; Igaku Tokei Kenkyujo, Inc., Ube, Japan). The analysis was based on orthopaedic surgeons in 2015 using propensity score analysis and matching by age and workplace setting. A Chi-square test was used to examine significant differences before and after matching in cross-analysis as well as to compare each item between 2015 and 2020. Statistical significance was set at a *p* value <0.05.

### 2.7. Required Sample Size

The required sample size was obtained using Stat Flex 6.0 (ver. 6.0; Igaku Tokei Kenkyujo Inc., Ube, Japan). The ratio of the control group was set to 0.5, the difference in the ratio to be detected was 0.25, the type 1 error rate was 0.05, the type 2 error rate was 0.20, and the control group: test group ratio was 1 : 1 with 60 cases in each group.

## 3. Results

### 3.1. Response Rate

In 2015, responses were received from physicians in their 30s–80s (median age: 50s). In 2020, we received responses from physicians in their 20s–80s (median age: 50s). Of the 600 respondents, 287 (47.8% response rate) responded (Table 1). Out of these, 74 individuals were orthopaedic surgeons in 2015, while the number increased to 282 in 2020.

### 3.2. Propensity Score Analysis

From the data collected in 2015 and 2020, only orthopaedic surgeons were selected and matched by physician age (years) and work type. Consequently, 74 patients were selected from each group, meeting the required sample size (Figure 1). In both groups, 11 participants were in their 30s, 25 in their 40s, 24 in their 50s, 10 in their 60s, 3 in their 70s, and 1 in their 80s (Table 2). In both groups, there were 17 doctors working in facilities with more than 200 beds, 15 doctors working in facilities with fewer than 200 beds, and 42 doctors who worked as general practitioners (Table 2).

### 3.3. Imaging Tests

In 2015, 45 respondents wanted a simple radiograph of the thoracolumbar spine, 30 respondents wanted a bone mineral density scan of the lumbar spine and hip joint, 4 respondents wanted a magnetic resonance imaging (MRI) scan, and 15 respondents did not need any additional tests (Table 3). Comparing 2015 with 2020, the proportion of physicians who indicated that a simple radiograph of the thoracolumbar spine and no further testing was needed decreased, whereas the number of cases in which they considered adding bone mineral density testing of the lumbar spine and hip joints increased (Table 3). For clinic-based doctors, the addition of bone mineral density testing did not change between 2015 and 2020. However, the number of hospital-based doctors requesting bone mineral density testing of the lumbar spine and hip extensively increased in 2020 (Table 4).

### 3.4. Blood Tests

**Figure 3:**
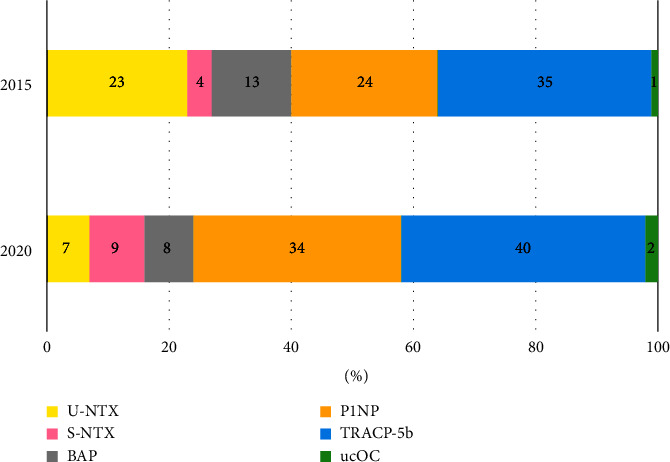
Selection of bone metabolic markers in 2015 and 2020. Urinary NTX decreased, and blood testable bone metabolic markers, such as serum NTX, BAP, P1NP, and TRACP-5b, increased. NTX: type I collagen cross-linked N-telopeptides, BAP: alkaline phosphatase, P1NP: procollagen I N-terminal propeptide, TRACP-5b: tartrate-resistant acid phosphatase 5b, ucOC: undercarboxylated osteocalcin.

With regards to the measurement of bone metabolic markers, the number of respondents who were tested for certain bone metabolic markers remained unchanged at 59 (79.7%) in 2015 and 60 (81.1%) in 2020. The breakdown of bone metabolic markers was as follows: urinary NTX (*n* = 22), serum NTX (*n* = 4), alkaline phosphatase (BAP; *n* = 12), procollagen I N-terminal propeptide (P1NP; *n* = 23), tartrate-resistant acid phosphatase 5b (TRACP-5b; *n* = 33), undercarboxylated (ucOC; *n* = 1), and deoxypyridinoline (DPD; *n* = 0) in 2015; in 2020, the number of physicians requesting urinary NTX was 9, 12 requested serum NTX, 11 requested BAP, 44 requested P1NP, 52 requested TRACP-5b, 3 requested ucOC, and 0 requested DPD (Table 3). Urinary NTX decreased and serum NTX, P1NP, and TRACP-5b levels increased (Figure 3). Notably, 19 orthopaedic surgeons indicated that they would measure 25(OH)D in 2020, compared to two who said they would (Table 3). This indicated a remarkable increase among both clinic- and hospital-based doctors (Table 4).

### 3.5. Therapeutic Drugs

**Figure 4:**
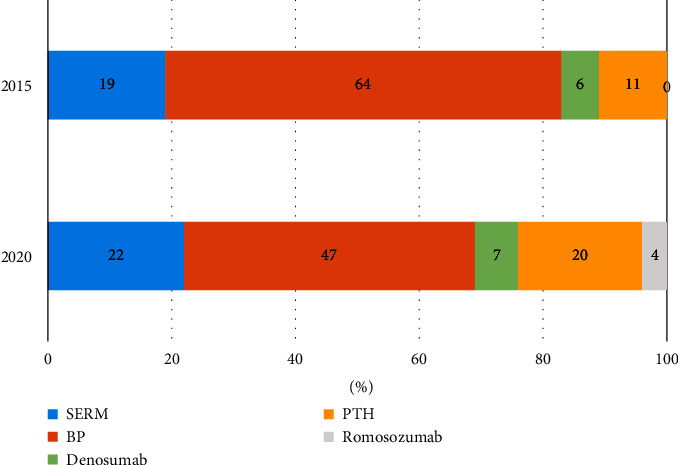
Selection of the main osteoporosis drugs in 2015 and 2020. The percentage of BP drugs showed a decreasing trend, whereas those of PTH and romosozumab showed an increasing trend. BP: bisphosphonate, PTH: parathyroid hormone.

In 2015, the drugs prescribed for this case were active vitamin D3 in 50 patients, SERM in 16 patients, bisphosphonate (BP) in 54 patients, denosumab in 5 patients, PTH in 9 patients, and vitamin K in 2 patients; in 2020, the drugs prescribed were active vitamin D3 in 59 patients, SERM in 18 patients, bisphosphonate in 39 patients, and denosumab in 6 patients (Figure 4). Between 2015 and 2020, bisphosphonate prescribing decreased, whereas denosumab, PTH, and romosozumab prescribing increased (Table 3).

### 3.6. Treatment Policy

In 2015, physicians' approaches for treatment were as follows: 43 physicians utilised treatment experience, 25 utilised osteoporosis-related guidelines, 16 utilised lectures, 6 utilised textbooks, 2 referred to work colleagues, and 2 used medical representatives (MRs) as their most important references in deciding on a treatment plan. Between 2015 and 2020, there was little to no change in the aforementioned resources.

## 4. Discussion

In this study, the 2015 and 2020 surveys were compared and matched for age and work type using propensity score analysis. We then surveyed physicians' attitudes towards testing and treatment for osteoporosis. Other surveys frequently inquire about patients' experiences with tests for osteoporosis, such as bone mineral density and radiography, whether they had ever been diagnosed with a fracture, as well as information about their lifestyle, exercise habits, and diet, such as vitamin D intake [6, 7]. Kim et al. surveyed physicians to determine their perceptions of problems associated with severe osteoporosis, the drugs used to treat the disease, the problems they experienced, and their treatment goals [8]. Our study involved a new approach in that we presented specific hypothetical cases and asked each physician what they would do in their actual clinical practice.

In terms of imaging studies, the percentage of respondents who indicated that they would test for the bone mineral density of the lumbar spine and hips extensively increased in 2020 compared with 2015. In particular, the percentage of hospitalists who indicated that they would test the mineral density of the lumbar spine and hip remarkably increased. The prevalence of machines that can measure bone mineral density of the lumbar spine and hip has been increasing [9, 10], and it is possible that the number of hospitals capable of measuring bone mineral density of the lumbar spine and hip has increased over the past five years. In addition, the 2015 guidelines for the prevention and treatment of osteoporosis recommend measuring the BMD of the lumbar spine and hip [11]. The bone mineral density of the radius is supposed to be used when the bone mineral densities of the lumbar spine and hip are difficult to measure [4]. However, it was assumed that many physicians desired to request bone density testing of the lumbar spine and hip, if feasible. In 2012, the vertebral fracture evaluation criteria were revised, and a quantitative evaluation method using lumbar spine radiographs was added. The importance of radiographic imaging is gradually becoming more widespread [12].

Bone metabolic markers can be used not only to study bone metabolic turnover but also as risk factors for fractures independent of bone mineral density [13]. They can also be used to predict the effects of bone-resorption inhibitors on bone mineral density [14]. Ishikawa et al. reported that the higher the bone metabolic markers, the higher the incidence of hypocalcaemia after denosumab administration and that the test can be linked to the early detection of adverse events [15]. Tsai et al. reported that there was a relationship between decreased P1NP and increased bone mineral density [16]. The 2018 revision of the Guide for the Appropriate Use of Bone Metabolic Markers in the Treatment of Osteoporosis, which clarified the treatment of bone metabolic markers, was also considered a reason for the increase in the number of selectors [17]. The International Osteoporosis Foundation and International Federation of Clinical Chemistry recommend serum P1NP and CTX-1 (Type I collagen cross-linked C-telopeptide) as reference markers of bone formation and resorption, respectively [18]. However, CTX-1 was not an option in this study. We aim to include this marker in our next survey. The percentage of urine NTX tested decreased from 29.7% to 12.2% for bone metabolic markers. Serum NTX has less diurnal variation than urine NTX but tends to be higher with decreased renal function [19, 20]. Because urine NTX requires measurement of the second urine sample early in the morning, if the test is to be performed strictly, it is necessary to give the patient a urine test container in advance [21]. Since BAP, P1NP, and TRACP-5b have fewer diurnal variations [20], physicians have started using these tests in their practices.

As per the therapeutic agents, BP prescriptions decreased and PTH and romosozumab prescriptions increased although there was no significant difference between 2015 and 2020. PTH and romosozumab can be used for osteoporosis with high fracture risk but not for normal osteoporosis [22]. The results of this study suggest a change in policy to select drugs that can increase bone mineral density at an earlier stage [23, 24] to reduce the risk of fracture.

The hypothetical case in the present study had diabetes and deteriorated bone quality [25]. The 10-year major osteoporotic fracture risk as assessed by FRAX was 20%. If the patient is under 75 years of age and has bone mineral density loss, treatment should be initiated when the 10-year major osteoporotic fracture risk is 15% or greater [26]. Considering the 1.6-fold increase in the risk of major osteoporotic fractures in the presence of diabetes mellitus, treatment should be initiated in this case.

Because the purpose of the survey was to investigate which other tests they would like to perform, some physicians felt that they did not have sufficient information on the case presented. As a result, some said that they would consider additional tests after seeing the test results or that they could not decide on the type of medication without seeing the test results, which is a limitation of the survey. It should also be noted that this was a survey of physicians' attitudes and was not conducted in actual practice. In practice, patients may not accept the suggestions of physicians. In the future, we intend to continue conducting the survey to address the discrepancies in examination content resulting from changes over time and variations among hospitals. Our aim is to foster collaboration between hospitals and clinics to establish a cohesive approach.

Understanding the differences in thinking between clinical- and hospital-based physicians could be used to augment collaboration between hospitals and clinics and between hospitals, including incorporating mainstream laboratory tests and avoiding duplication of testing.

A limitation of this study is that the results were obtained from a questionnaire limited to orthopaedic surgeons in urban areas of Japan and may not apply to doctors in other countries or specialties other than orthopaedic surgery. Because this is a questionnaire survey of hypothetical cases, it is possible that the actual practice may differ. In addition, because the respondents were asked to choose from a list of options rather than fill in their answers, minority opinions could not be addressed. Romosozumab became available in Japan in March 2019. Therefore, as of 2015, when the first questionnaire was conducted, romosozumab was not available. The use of bone metabolism markers as monitoring in drug therapy is controversial, as the currently commercially available methods for measuring bone metabolism markers are imprecise [27]. Meanwhile, in Japan, the Japanese Osteoporosis Society recommends the use of bone metabolism markers [17]. The purpose of the present questionnaire survey was to ascertain the use of bone metabolism markers and not to recommend their use.

## 5. Conclusions

A survey comparing 2015 and 2020 was conducted to assess osteoporosis testing and treatment strategies among orthopaedic surgeons. In 2020, a greater percentage of surgeons reported conducting bone mineral density measurements at the lumbar spine and hip compared to 2015. Moreover, there was an increase in the number of physicians choosing bone metabolic markers that exhibit minimal diurnal variation and can be measured through blood tests. In addition, more physicians were investigating potential causes of osteoporosis, such as 25(OH)D levels. Notably, there was a marked trend towards administering drugs with the potential to increase bone mineral density at an earlier stage of treatment. The selection of test items and treatment methods had changed over time.

## Figures and Tables

**Table 1:** Composition of the number of people before matching by the propensity score.

		2015	2020	*p* value
Total number of doctors		143	287	

Age	20s (%)	0 (0)	14 (4.9)	*p* < 0.05
	30s (%)	11 (7.7)	51 (17.8)	
	40s (%)	42 (29.4)	57 (19.9)	
	50s (%)	46 (32.2)	91 (31.7)	
	60s (%)	33 (23.1)	50 (17.4)	
	70s (%)	8 (5.6)	16 (5.6)	
	80s (%)	3 (2.1)	8 (2.8)	

Workplace setting	Healthcare institutions with more than 200 beds (%)	17 (11.9)	102 (35.5)	*p* < 0.001
	Healthcare institutions with less than 200 beds (%)	16 (11.2)	50 (17.4)	
	General practitioner (%)	110 (76.9)	134 (46.7)	
	Non-response	0 (0)	1 (0.3)	

Major specialty department	Orthopaedic surgery	74 (51.7)	282 (98.3)	*p* < 0.001
	Internal medicine	60 (42.0)	3 (1.0)	
	Surgical department	4 (2.8)	0 (0)	
	Obstetrics and gynaecology	2 (1.4)	0 (0)	
	Neurosurgery	1 (1.0)	0 (0)	
	Pain clinic	2 (1.4)	0 (0)	
	Rehabilitation	0 (0)	2 (0.7)	

Statistics were a Chi-square test.

**Table 2:** Composition of headcount after matching by the propensity score (age, type of work, and primary advocate department).

		2015	2020	*p* value
The number of doctors extracted		74	74	

Age	30s (%)	11 (14.9)	11 (14.9)	1.00
	40s (%)	25 (33.8)	25 (33.8)	
	50s (%)	23 (31.1)	23 (31.1)	
	60s (%)	11 (14.9)	11 (14.9)	
	70s (%)	3 (4.1)	3 (4.1)	
	80s (%)	1 (1.4)	1 (1.4)	

Workplace setting	Healthcare institutions with more than 200 beds (%)	17 (23.0)	17 (23.0)	1.00
	Healthcare institutions with less than 200 beds (%)	15 (20.3)	15 (20.3)	
	General practitioner (%)	42 (56.8)	42 (56.8)	

Major specialty department	Orthopaedic surgery (%)	74 (100)	74 (100)	1.00

Statistics were a Chi-square test.

**Table 3:** Postmatching test items, prescription details, and knowledge acquisition methods.

		2015	2020	*p* value
The number of doctors extracted		74	74	

Examination Items^*∗*^	Xp of the thoracolumbar spine (%)	45 (60.8)	38 (51.4)	0.25
	Bone mineral density of the lumbar spine and hip joint (%)	30 (40.5)	47 (63.5)	*p* < 0.01
	MRI of spine (%)	4 (5.4)	2 (2.7)	0.40
	No further examination (%)	15 (20.3)	8 (10.8)	0.11

Breakdown of bone metabolic markers^*∗*^	Any bone metabolic markers (%)	59 (79.7)	60 (81.1)	0.84
	Urinary NTX (%)	22 (29.7)	9 (12.2)	*p* < 0.01
	Serum NTX (%)	4 (5.4)	12 (16.2)	*p* < 0.05
	BAP (%)	12 (16.2)	11 (14.9)	0.82
	P1NP (%)	23 (31.1)	44 (59.5)	*p* < 0.001
	TRACP-5b (%)	33 (44.6)	52 (70.3)	*p* < 0.01
	ucOC (%)	1 (1.4)	3 (4.1)	0.31
	DPD (%)	0 (0)	0 (0)	1.00

Blood test	25(OH)D	2 (2.7)	19 (26.7)	*p*<0.001

Treatment^*∗*^	Active vitamin D3 (%)	50 (67.6)	59 (79.7)	0.09
	SERM (%)	16 (21.6)	18 (24.3)	0.70
	Bisphosphonate (%)	54 (73.0)	39 (52.7)	0.11
	Denosumab (%)	5 (6.8)	6 (8.1)	0.75
	PTH preparation (%)	9 (12.2)	17 (23.0)	0.08
	Romosozumab (%)	0 (0)	3 (4.1)	0.08
	Vit K	2 (2.7)	0 (0)	0.15

Exercise therapy	Exercise therapy (%)	37 (50.0)	41 (55.4)	0.51

Nutritional guidance	Nutritional guidance (%)	26 (35.1)	32 (43.2)	0.31

Knowledge^*∗*^	Treatment experience (%)	43 (58.1)	40 (54.1)	0.62
	Guidelines (%)	25 (33.8)	24 (32.4)	0.86
	Lectures (%)	16 (21.6)	19 (26.7)	0.56
	Textbooks (%)	6 (8.1)	5 (6.8)	0.75
	Colleagues (%)	2 (2.7)	1 (1.4)	0.56
	Medical representative (%)	2 (2.7)	1 (1.4)	0.56
	No response (%)	0 (0)	2 (2.7)	0.15

Statistical analysis included a Chi-square test. Xp: X-ray photography; MRI: magnetic resonance imaging, NTX: type I collagen cross-linked N-telopeptide; BAP: alkaline phosphatase; P1NP: procollagen I N-terminal propeptide; TRACP-5b: tartrate-resistant acid phosphatase 5b; PTH: parathyroid hormone; ucOC: undercarboxylated osteocalcin; DPD: deoxypyridinoline. ^*∗*^Duplicate responses were obtained.

**Table 4:** Hospital-based and clinic-based orthopaedic surgeons, 2015 and 2020.

*Hospital-based orthopaedic surgeon*			
Year in which the survey was conducted	2015	2020	*p* value

Number of doctors	32	32	
BMD measurement of the lumbar spine or hip joint (%)	18 (56.3)	27 (84.4)	*p* < 0.05
Xp of thoracolumbar spine (%)	19 (59.4)	16 (50.0)	0.45
25(OH)D (%)	0 (0)	8 (25.0)	*p* < 0.01
Bone metabolic markers (%)	25 (78.1)	30 (93.8)	0.07
Therapeutic exercise (%)	16 (50.0)	15 (46.9)	0.80
Nutritional guidance (%)	11 (34.4)	13 (40.6)	0.61

*Orthopaedic surgeons working in clinics*			
Year in which the survey was conducted	2015	2020	*p* value

Number of doctors	42	42	
BMD measurement of the lumbar spine or hip joint (%)	12 (28.6)	20 (47.6)	0.07
Xp of thoracolumbar spine (%)	26 (61.9)	22 (52.4)	0.38
25(OH)D (%)	2 (5.8)	11 (26.2)	*p* < 0.01
Bone metabolic markers (%)	34 (81.0)	30 (71.4)	0.31
Therapeutic exercise (%)	21 (50.0)	25 (59.5)	0.38
Nutritional guidance (%)	15 (35.7)	19 (45.2)	0.37

Statistics were a Chi-square test. BMD, bone mineral density; Xp, X-ray photography; 25(OH)D,25-hydroxyvitamin D.

## Data Availability

The datasets used and analysed to support the findings of this study are available from the corresponding author upon reasonable request.
